# Peptide-Purified Anti-N-methyl-D-aspartate Receptor (NMDAR) Autoantibodies Have Inhibitory Effect on Long-Term Synaptic Plasticity

**DOI:** 10.3390/ph17121643

**Published:** 2024-12-06

**Authors:** Charlotte Day, John-Paul Silva, Rebecca Munro, Brice Mullier, Véronique Marie André, Christian Wolff, Gary J. Stephens, Angela Bithell

**Affiliations:** 1 School of Pharmacy, University of Reading, Whiteknights, Reading RG6 6AJ, UK; charlotte.day7@outlook.com; 2 UCB Pharma, 208 Bath Road, Slough SL1 3WE, UK; john.silva@ucb.com (J.-P.S.); rebecca.munro@ucb.com (R.M.); 3 UCB Pharma, Chemin du Foriest, 1420 Braine l’Alleud, Belgium; brice.mullier@ucb.com (B.M.); veroniquemarie.andre@ucb.com (V.M.A.); christian.wolff@ucb.com (C.W.)

**Keywords:** NMDA receptor autoantibodies, hippocampal neuron, long-term potentiation, multi-electrode arrays

## Abstract

**Background/Objectives:** Recent studies, typically using patient cerebrospinal fluid (CSF), have suggested that different autoantibodies (Aabs) acting on their respective receptors, may underlie neuropsychiatric disorders. The GluN1 (NR1) subunit of the N-methyl-D-aspartate receptor (NMDAR) has been identified as a target of anti-NMDAR Aabs in a number of central nervous system (CNS) diseases, including encephalitis and autoimmune epilepsy. However, the role or the nature of Aabs responsible for effects on neuronal excitability and synaptic plasticity is yet to be established fully. **Methods:** Peptide immunisation was used to generate Aabs against selected specific GluN1 extracellular sequences based on patient-derived anti-NMDAR Aabs that have been shown to bind to specific regions within the GluN1 subunit. ‘Protein A’ purification was used to obtain the total IgG, and further peptide purification was used to obtain a greater percentage of NMDAR-target specific IgG Aabs. The binding and specificity of these anti-NMDAR Aabs were determined using a range of methodologies including enzyme-linked immunosorbent assays, immunocytochemistry and immunoblotting. Functional effects were determined using different in vitro electrophysiology techniques: two-electrode voltage-clamps in *Xenopus* oocytes and measures of long-term potentiation (LTP) in ex vivo hippocampal brain slices using multi-electrode arrays (MEAs). **Results:** We show that anti-NMDAR Aabs generated from peptide immunisation had specificity for GluN1 immunisation peptides as well as target-specific binding to the native protein. Anti-NMDAR Aabs had no clear effect on isolated NMDARs in an oocyte expression system. However, peptide-purified anti-NMDAR Aabs prevented the induction of LTP at Schaffer collateral-CA1 synapses in ex vivo brain slices, consistent with causing synaptic NMDAR hypofunction at a network level. **Conclusions:** This work provides a solid basis to address outstanding questions regarding anti-NMDAR Aab mechanisms of action and, potentially, the development of therapies against CNS diseases.

## 1. Introduction

In the CNS, NMDARs are one of the main ionotropic glutamate receptors, alongside α-amino-3-hydroxy-5-methyl-4-isoxazolepropionic acid (AMPA) and kainate receptors, and have critical functions in synaptic plasticity and learning and memory [1]. NMDARs are typically heterotetramers comprising two GluN1 subunits present in all NMDARs (encoded by *GRIN1*, although with a number of splice variants) and two GluN2 subunits, with four different sub-types (GluN2A-D, encoded by *GRIN2A-D*, respectively) that exhibit different spatio-temporal expressions and functional properties. A third subunit type, GluN3, has two subtypes (GluN3A and GluN3B, encoded by *GRIN3A* and *GRIN3B*, respectively), which also exhibit different spatiotemporal expressions and functional properties (reviewed in [2]). Autoantibodies (Aabs) produced by the body against self-neurotransmitter receptors including NMDARs have been implicated in CNS diseases. Anti-NMDAR Aabs of the IgG class directed against the GluN1 (NR1) subunit have been found in the CSF of patients with anti-NMDAR encephalitis (ANRE), a condition characterised by seizures, psychosis and cognitive deficits [3], and in patients with forms of autoimmune epilepsy [4,5]. Such evidence sparked interest in investigating the functionality of anti-NMDAR Aabs [6]. For example, an early study showed that post-mortem hippocampus from ANRE patients expressed significantly less NMDARs than age-matched controls [7]. The consensus view is that anti-NMDAR Aabs in patient brains can induce pathological changes leading to symptoms. It has been shown that immunisation with conformationally stabilised NMDA holoreceptors in liposomes generates anti-NMDAR Aabs and causes neuroinflammation [8]. However, it is worth noting that several studies, including recent in vivo animal studies using immunisation with NMDAR peptides, suggest a current lack of evidence that anti-NMDAR Aabs alone can initially induce encephalitis but might rather modulate the inflammatory response [9,10]. It has also been shown that the in vivo infusion of ANRE patient CSF, containing anti-NMDAR Aabs, resulted in increased chemical-induced epileptic seizures, behavioural changes, and memory impairment in rodents [11,12,13]. Electroencephalogram recordings of mice infused intraventricularly with ANRE patient CSF showed a higher frequency of seizures [14]. At a cellular level, the exposure of ANRE patient CSF to primary hippocampal neurons in vitro resulted in a reduction in NMDAR currents [7,15]; monoclonal antibodies cloned from an ANRE patient caused similar effects [16,17]. A primary mechanism, originally proposed by Hughes et al. (2010), was that treatment with patients’ Aabs bound to and cross-linked NMDARs, leading to a selective decrease in synaptic NMDAR currents via receptor internalisation [7]. By contrast, a recent study generated expression-purified IgGs from a range of monoclonal antibodies produced in mice immunised with intact NMDAR GluN1/GluN2A protein [18]; one such species (termed IgG2) produced a rapid block of NMDAR currents, both in oocytes and in a mammalian HEK cell expression system. These anti-NMDAR Aab species were proposed to function via the allosteric downregulation of the NMDAR function due to the stabilisation of an inactive NMDAR confirmation [18]. It was also reported that Aabs have the ability to activate the complement ‘membrane attack complex’ in NMDAR-expressing cells, proposed to be responsible for some symptoms observed in ANRE patients [19]. Thus, the rationale of the present study was to further investigate the mechanisms underlying the potential pathophysiology with different purified anti-NMDAR Aabs. Peptide-purified anti-NMDAR Aabs had no clear effects on NMDAR gating in an in vitro oocyte expression system but showed an inhibitory effect on long-term synaptic plasticity in ex vivo hippocampal brain slices. Increasing our mechanistic knowledge of how NMDAR Aabs cause effects in the CNS may lead to a better understanding/treatment of diseases such as ANRE and autoimmune epilepsies.

## 2. Results

### 2.1. Generation and Characterisation of NMDAR Aabs

**Figure 1:**
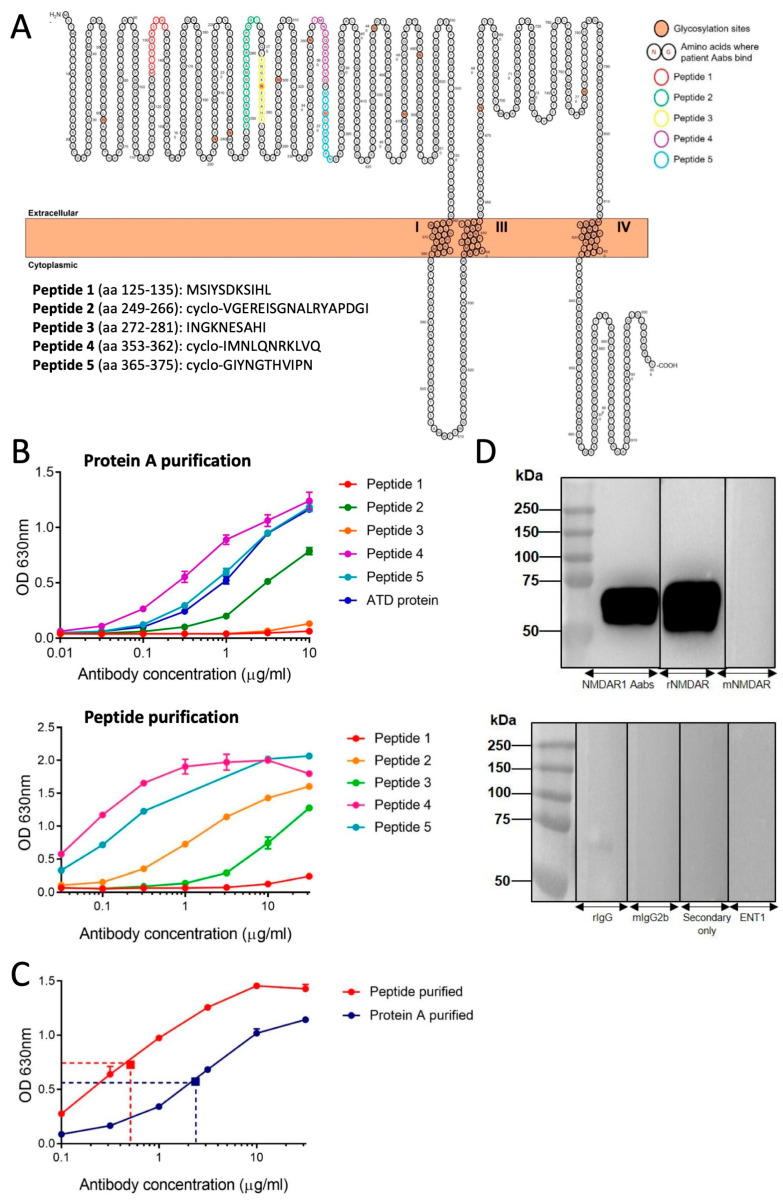
Purification of NMDAR Aabs: (**A**) NMDAR peptide sequences used for immunisation. All peptides were located within the amino terminal domain (ATD) of the GluN1 (NR1) subunit and generated with the addition of a C residue and an Ac residue on either end. Peptides 2, 4 and 5 were cyclised via a thioester to help better represent the true epitope. (**B**) ELISA for Protein A-purified (upper panel) and peptide-purified (lower panel) anti-NMDAR Aabs; in particular, peptides 2, 4 and 5 exhibited robust binding following further peptide purification. (**C**) ELISA for peptide-purified vs. Protein A-purified Aabs. Dotted lines represent 50% reduction in signal values. Each n = 3 technical replicates per concentration. (**D**) Human GluN1 subunit ATD probed with anti-NMDAR Aabs (upper panel) and controls rIgG, mIgG2b, secondary only and ENT1. Blots incubated with anti-NMDAR Aabs detected a strong band at approximately 60 kDa, the expected size of the ATD. Representative blot selected from n = 3 technical replicates.

Five immunisation peptides were generated based on NMDAR GluN1 (NR1) subunit sequences targeted by Aabs identified from patient CSFs or sera (Figure 1A). Following immunisation with all five peptides, the resulting sera were Protein A-, and then peptide-, purified (see Section 4). Terminal serum titres were measured by ELISA following Protein A and peptide purification (Figure 1B). A comparative ELISA demonstrated increased anti-NMDAR Aab binding to the amino terminal domain (ATD) protein following peptide purification (Figure 1C). Thus, peptide-purified material required 0.51 µg/mL to elicit a 50% maximal concentration response, whereas the Protein A-purified material required 2.36 µg/mL to elicit a 50% maximal response, indicating an increase in binding to ATD protein due to a greater percentage of NMDAR-target specific IgG Aabs. The specificity of peptide-purified anti-NMDAR Aabs for the ATD of the GluN1 subunit was determined by Western blot, with a strong band at the predicted ATD size of approximately 60 kDa pre- and post-peptide purification, similar to a commercial anti-GluN1 antibody that recognises the ATD (rabbit polyclonal anti-GluN1 antibody (rNMDAR), Synaptic Systems, Cat no. 114 103, Figure 1D). Conversely, no band was observed with a commercial anti-GluN1 (NR1) antibody that does not target the ATD (mouse monoclonal anti-GluN1 antibody (mNMDAR), Synaptic Systems, Cat no. 114 011) or negative control antibodies (rIgG, mIgG2b, secondary only or an unrelated primary, equilibrative nucleoside transporter 1 (ENT1), Figure 1D).

**Figure 2:**
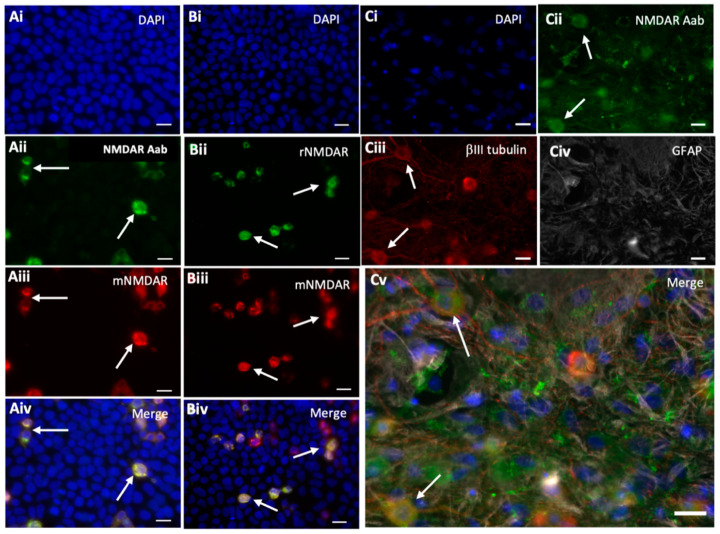
Peptide-purified anti-NMDAR Aabs show selective immunocytochemical staining. NMDAR GluN1 (NR1)-transfected HEK cells were stained with peptide-purified anti-NMDAR Aabs (1:100), plus one of two commercial anti-GluN1 antibodies: mouse anti-GluN1 (mNMDAR, 1:100), rabbit anti-GluN1 (rNMDAR, 1:100) and a nuclear stain (DAPI, 1:10,000, blue). (**Ai**–**Aiv**) Cells transfected with GluN1 were detected by anti-NMDAR1 Aabs (**Aii**), which were co-labelled by the commercial antibody mNMDAR (**Aiii**), (as shown by white arrows). (**Bi**–**Biv**) Both commercial anti-GluN1 antibodies rNMDAR (**Bii**) and mNMDAR (**Biii**) co-labelled the same GluN1-transfected cells (as shown by white arrows). Representative images selected from n = 3 biological replicates. (**Ci**–**Cv**) Primary cortical neuronal cells (DIV14) were stained with anti-NMDAR Aabs (1:100, green, **Cii**); co-stained with the neuronal marker βIII tubulin (1:500, red, **Ciii**); the astrocytic marker GFAP (1:400, **Civ**) and DAPI (1:10,000, blue, **Cv**). Representative images selected from n = 3 biological replicates. Scale bar = 20 µm throughout.

Human embryonic kidney 293 (HEK) cells were transfected with a vector encoding the GluN1 subunit. Immunocytochemistry performed using peptide-purified anti-NMDAR Aabs and the commercial mNMDAR antibody showed positively co-labelled GluN1-transfected HEK cells (Figure 2Ai–Aiv). The commercial rNMDAR antibody showed similar co-labelling (Figure 2Bi–Biv). No positively labelled cells were observed with the class-specific negative control rIgG and secondary-only controls in GluN1-transfected HEK cells or with GluN1 Aabs or commercial anti-GluN1 antibodies in empty vector-transfected HEK cells (Supplemental Figure S1). To confirm the presence of anti-NMDAR Aabs in neurons, immunocytochemistry was also performed in primary cortical neurons, where anti-NMDAR Aabs labelling colocalized with the neuronal marker βIII tubulin but not with the astrocyte marker GFAP (Figure 2Ci–Cv). Overall, further peptide purification resulted in more concentrated NMDAR GluN1 Aabs, which showed specific labelling in both GluN1-transfected HEK cells and in primary neurons.

### 2.2. Lack of Functional Effects of NMDAR Aabs in GluN1/GluN2A Expressing Xenopus Oocytes

**Figure 3:**
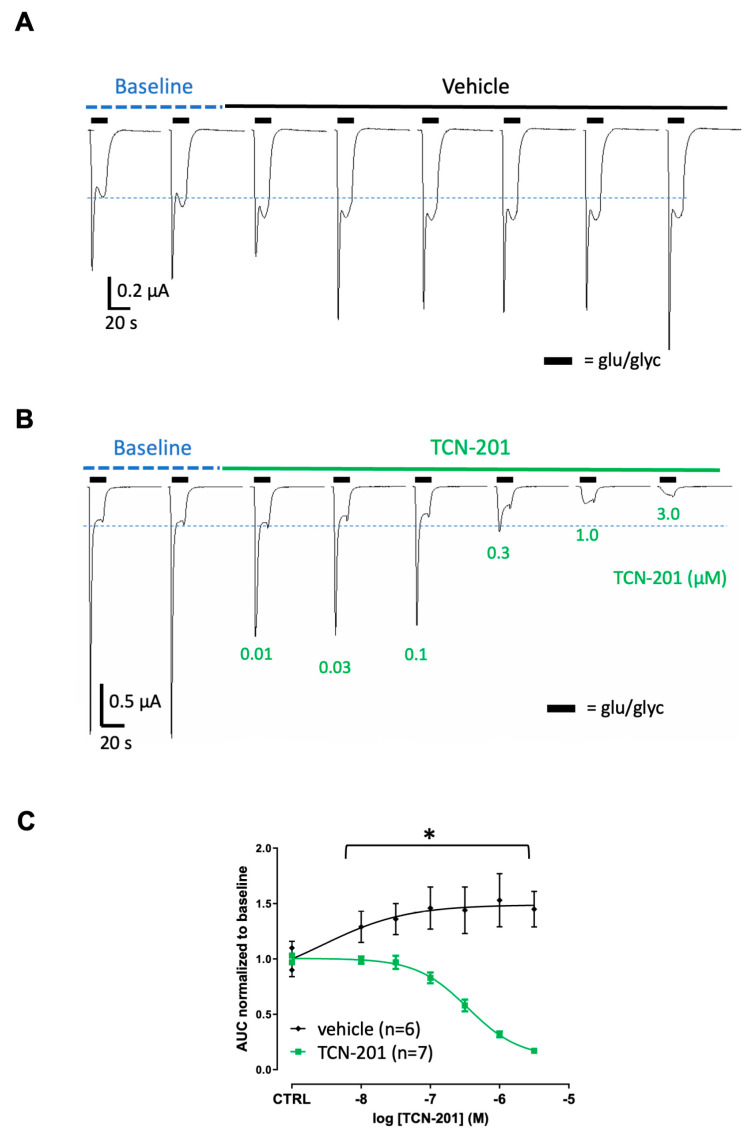
NMDAR negative modulator significantly reduces NMDAR currents in *Xenopus* oocytes: (**A**) Traces of NMDAR-evoked responses over time: 1 µM of glutamate/10 µM of glycine-induced NMDAR currents were elicited every 3 min. The last 2 baseline traces of 4 traces are depicted and were used to normalise to the control before switching to the vehicle (control). (**B**) Traces of NMDAR-evoked responses before and after the addition of increasing concentrations of TCN-201. (**C**) Graph shows a significant reduction in the normalised glu/gly-evoked (AUC) response with the increasing concentration of TCN-201 vs. vehicle control. A two-way ANOVA with Dunnett’s multiple comparisons revealed both a significant effect of drug vs. vehicle (* = *p* < 0.0001) and drug concentration used (* = *p* < 0.0001), respectively). Data are represented as mean ± SD. Each oocyte was used for the baseline and treatment throughout the 30 min experiment.

**Figure 4:**
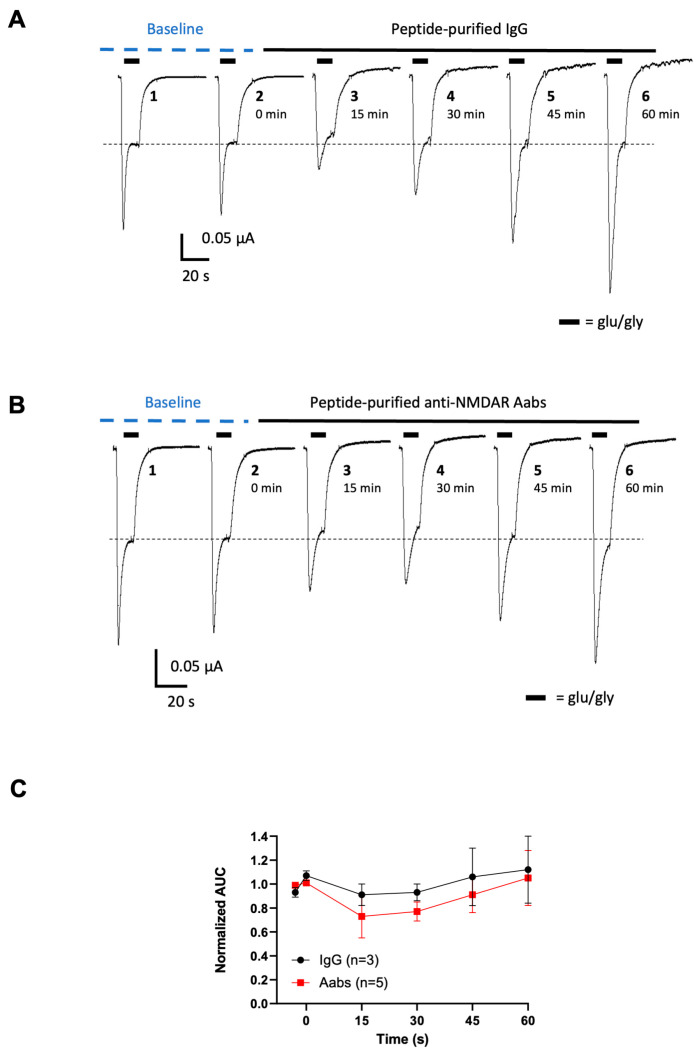
Peptide-purified anti-NMDAR Aabs have no effect on NMDAR current in *Xenopus* oocytes: (**A**) Traces of NMDAR-evoked responses over time before and after incubation with control peptide-purified IgG: 10 µM of glutamate/10 µM of glycine-induced NMDAR currents were elicited every 15 min in the presence of IgG (1:300 dilution) applied for up to 60 min. (**B**) Traces of NMDAR-evoked responses over time before and after the incubation of peptide-purified anti-NMDAR Aabs: 10 µM of glutamate/10 µM of glycine-induced NMDAR currents were elicited every 15 min in the presence of anti-NMDAR Aabs (1:300 dilution) applied for up to 60 min. (**C**) Graph shows effects of peptide-purified anti-NMDAR Aabs and rIgG glutamate-evoked (AUC) responses normalised to the baseline (mean of applications 1 and 2 shown in (**A**,**B**)). There was no significant change in the AUC when compared to the baseline in anti-NMDAR Aabs or IgG incubated oocytes. All data are represented as mean ± SD. Each oocyte was used for the baseline and treatment throughout the 60 min experiment.

NMDAR GluN1/GluN2A subunit-expressing *Xenopus* oocytes were used to investigate effects of anti-NMADR Aabs on NMDAR currents [20,21]. The NMDAR negative allosteric modulator (TCN-201) was used as a positive control to confirm inhibition of the currents (Figure 3). TCN-201 (0.01–3.0 µM) reduced glutamate/glycine (glu/gly)-evoked NMDAR currents in oocytes (n = 6–7) in a concentration-dependent manner (Figure 3B,C) with a maximal inhibition of >90% at 3 µM. The half-maximal inhibitory concentration IC_50_ value for TCN-201 was 0.35 µM, in line with reported values [22]; these ligand data confirm the correct NMDAR expression in this system. In the vehicle-incubated oocytes, NMDAR currents did not decrease with time and even slightly increased with time when measuring the area under the curve (AUC) (Figure 3A,C). Peptide-purified anti-NMDAR Aabs or rIgG controls were then assessed in oocytes at 1:300 dilution (4 µg/mL, determined by ELISA, see Figure 1). Figure 4 shows results for peptide-purified anti-NMDAR Aabs (Figure 4B) and control peptide-purified IgGs (Figure 4A). There was no significant difference between NMDAR currents induced by the glu/gly application in oocytes incubated with IgGs or anti-NMDAR Aabs (Figure 4C). Of further note, Protein A-purified anti-NMDARs were similarly without effects compared to control Protein A-purified IgGs on NMDAR currents (Supplemental Figure S2). Overall, anti-NMDAR Aabs had no effect on NMDAR currents in oocyte experiments.

### 2.3. Schaffer Collateral-CA1 LTP Is Inhibited by Peptide-Purified Anti-NMDAR Aabs

**Figure 5:**
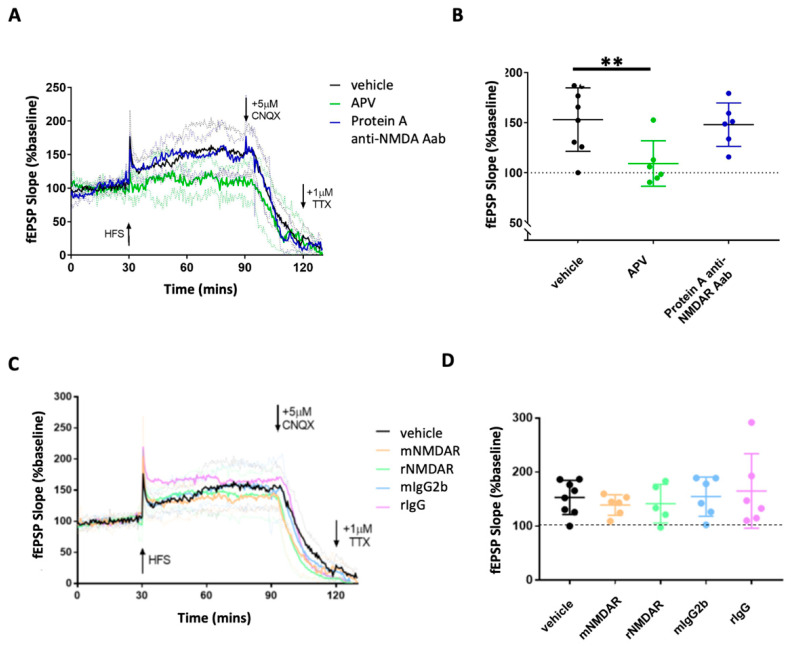
Effects of Protein A-purified anti-NMDAR Aabs and control antibodies on HFS-induced LTP in hippocampal brain slices: (**A**) Normalised mean traces of vehicle, 50 µM of APV and Protein A-purified anti-NMDAR Aab-treated slices undergoing LTP induction. Vehicle and Protein A-purified anti-NMDAR Aabs typically elicited a potentiation of ~150% post-HFS, which was maintained for at least 1 h. Greyed-out traces represent SD for each condition. The addition of APV almost completely inhibited any HFS-induced LTP. The addition of the AMPAR blocker CNQX (5 µM) caused a rapid reduction in HFS-induced LTP. Subsequent additions of the general voltage-gated sodium channel blocker, tetrodotoxin (TTX, 1 µM), abolished any remaining signal. (**B**) The comparison of LTP magnitudes (mean fEPSP slope during an 80–90 min application) revealed a significant reduction in the potentiation of APV-treated slices compared to the vehicle (*p* = 0.0046, n = 6–9 per group), whereas Protein A-purified anti-NMDAR Aab-treated slices revealed no significant changes in the potentiation of any condition when compared to vehicle slices (*p* = 0.98, n = 6–9 per group); one-way ANOVA with Dunnett’s multiple comparisons. Data are represented as mean ± SD, **: *p* < 0.01. (**C**) Normalised mean traces of vehicle, ‘positive’ (mNMDAR, rNMDAR) and ‘negative’ (mIgG2b, rIgG) control-treated slices during LTP induction. Greyed-out traces represent SD for each condition. Vehicle experiments typically elicited a potentiation of ~150% post-HFS, which was maintained for at least 1 h. (**D**) Comparison of the LTP magnitude (mean fEPSP slope during 80–90 min of the experiment) revealed no significant changes in the potentiation of any condition when compared to vehicle slices (n = 5–8 per group); one-way ANOVA was conducted with Dunnett’s multiple comparisons. Data are represented as mean ± SD.

**Figure 6:**
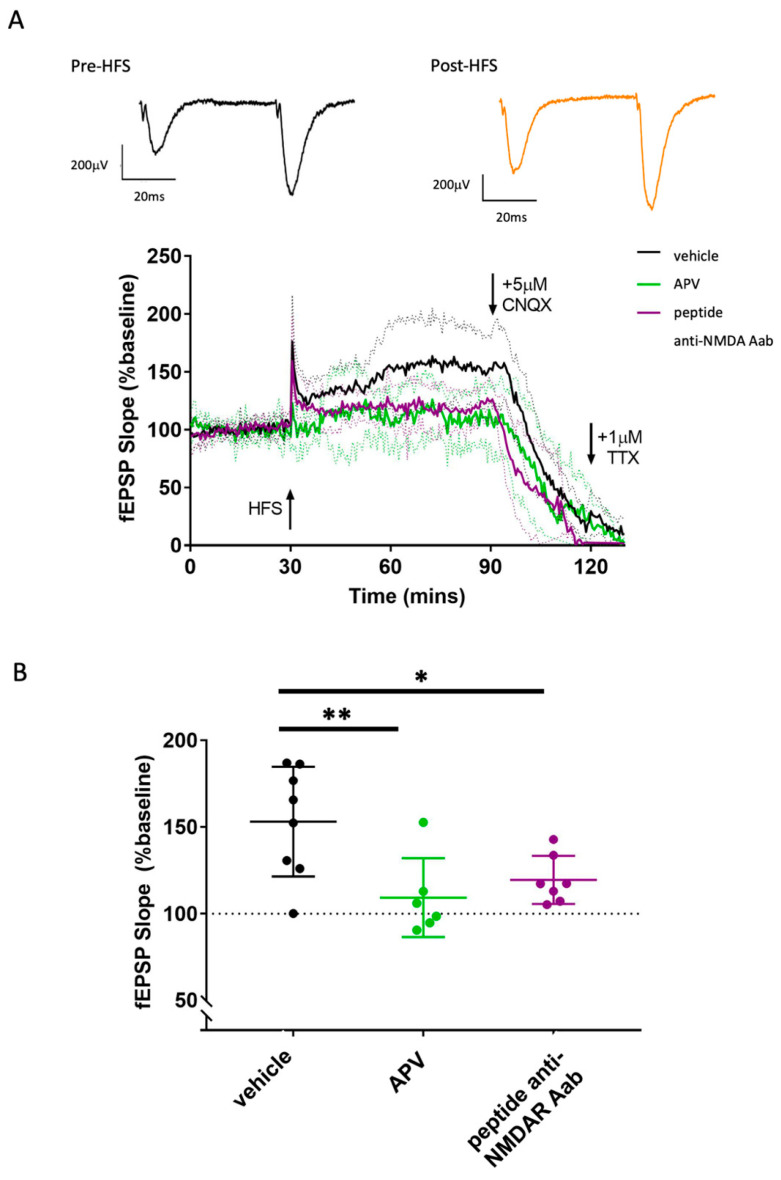
Peptide-purified anti-NMDR Aabs inhibit HFS-induced LTP in hippocampal brain slices: (**A**) Normalised mean traces of HFS-induced LTP for the vehicle, peptide-purified anti-NMDAR Aabs (1:1000, 1 h pre-incubation) or 50 µM of APV-treated slices. HFS elicited a potentiation of ~150% in vehicle conditions, which was maintained for at least 1 h. Greyed-out traces represent SD for each condition. The addition of APV dramatically inhibited HFS-induced LTP. Similarly, anti-NMDAR Aabs inhibited HFS-induced LTP. Inset shows representative traces of paired pulse recordings post-HFS (in vehicle). The addition of the AMPAR blocker CNQX (5 µM) caused a rapid reduction in HFS-induced LTP. Subsequent additions of the general voltage-gated sodium channel blocker, tetrodotoxin (TTX, 1 µM), abolished any remaining signal. (**B**) HFS-induced LTP (measured as the mean fEPSP slope increase over baseline) revealed a significant reduction in APV-treated (n = 6) slices vs. vehicle (n = 8), with anti-NMDAR Aab-treated slices (n = 7), demonstrating a similar significant reduction vs. the vehicle. Data are represented as mean ± SD; one-way ANOVA was conducted with Dunnett’s multiple comparisons * = *p* < 0.05, ** = *p* < 0.01.

We next investigated the effects of anti-NMDAR Aabs on NMDAR-dependent synaptic plasticity using MEA electrophysiology on ex vivo mouse hippocampal brain slices to record Schaffer collateral-CA1 LTP. A high-frequency stimulation (HFS) protocol was used to induce LTP, identified by a potentiation in the evoked field excitatory postsynaptic potential (fEPSP) slope for at least 1 h post-HFS in vehicle conditions with data normalised to pre-LTP baselines (Figure 5 and Figure 6A). Vehicle-treated slices generated a consistent HFS-induced potentiation at 1 h: 153.1 ± 31.6% (n = 8), which was significantly inhibited by the non-competitive NMDAR antagonist, APV (50 µM, 109.2 ± 22.7% (n = 6)), *p* < 0.01 compared to the vehicle; one-way ANOVA was conducted with Dunnett’s multiple comparisons (Figure 5A,B). Protein A-purified anti-NMDAR Aabs had no effect on HFS-induced potentiation compared to vehicle controls (Figure 5A,B). Using the same protocol, we also tested two commercial anti-GluN1 antibodies (rNMDAR and mNMDAR) and their class-specific negative controls (rIgG and mIgG2b, respectively), all at 1:1000 dilution. In all cases, no significant difference was observed in HFS-induced potentiation compared to vehicle controls (Figure 5C,D). By contrast to Protein A-purification, HFS-induced potentiation was significantly reduced in slices pre-incubated for 1 h with peptide-purified anti-NMDAR Aabs ((1:1000) 119.5 ± 13.8% (n = 7), *p* < 0.05) compared to the vehicle; one-way ANOVA was conducted with Dunnett’s multiple comparisons (Figure 6A,B). This level of inhibition was similar to that seen with APV. These data demonstrate a significant effect of the peptide-purified anti-NMDAR Aabs on the NMDAR function in an ex vivo brain slice model. In summary, peptide-purified anti-NMDAR Aabs were able to functionally inhibit LTP to a similar extent to APV, but equivalent effects were not observed with Protein A purified Aabs or the two commercial anti-GluN1 antibodies tested.

## 3. Discussion

Here, we show that peptide-purified anti-NMDAR Aabs generated through peptide immunisation bind to native NMDARs and inhibit LTP in mouse hippocampal brain slices, similar to those effects reported in studies using CSF or, in some studies, using monoclonal antibodies from ANRE patients. We were unable to demonstrate clear effects of our anti-NMDAR Aabs on NMDARs in a validated oocyte expression system.

### 3.1. Generation of Anti-NMDA Aabs as an Experimental Tool

The NMDAR GluN1 (NR1) subunit is obligatory for a fully functioning receptor [23]. Our strategy was to use immunising peptides based on five different human GluN1 subunit extracellular loop regions predicted to be of high immunogenicity to generate anti-NMDAR Aabs able to bind to the GluN1 subunit in its natural conformation. One of our peptides (cyclo-GIYNGTHVIPN) contained amino acids N368/G369; these residues, near the hinge region within the ATD, were shown to be crucial for human Aab recognition and binding to NMDARs from ANRE patient CSF [24] and to GluN1 monoclonal antibodies [16]. 

The majority of studies investigating effects of anti-NMDAR Aabs use material derived from patient CSF. Whilst these studies are of clear value, Aabs present in these mixed component preparations represent heterogenous families, targeted against different epitopes, including alternative receptor subtypes that may not contribute to functional effects of interest; overall, this makes the definitive assignment of function uncertain. In the present study, we use a strategy that involves a peptide purification step additional to a Protein A purification methodology. We demonstrate that additional purification produces anti-NMDAR Aabs with improved target binding compared to Protein A purification, indicating a greater percentage of NMDAR target-specific IgG Aabs. Of note here is that NMDAR Aabs that were only Protein A-purified had a statistically insignificantly effect on HFS-induced potentiation, whilst additional peptide purification produced anti-NMDAR Aabs that functionally inhibited LTP. Together, these data argue that the purification state of Aabs investigated here is of fundamental importance and that the peptide-purified anti-NMDAR Aabs represent useful experimental tools.

### 3.2. Functional Effects in Oocytes 

Overall, our results indicate that anti-NMDAR Aabs had no significant effect on NMDAR currents in GluN1/GluN2A-expressing oocytes following a 60 min incubation. Although there was some variability during the incubation, the same variability was seen in the rIgG control and in anti-NMDAR Aab-treated oocytes. These data confirm the lack of effect of anti-NMDAR antibodies on NMDAR function in oocytes. A previous study on oocytes has reported that, in the presence of sera from both ill and healthy patients or a commercial anti-GluN1 antibody (mNMDAR, as used here), NMDAR currents remained stable; whilst, in the presence of sera from seronegative patients, the NMDA current amplitude increased, leading authors to conclude that seropositive sera lowered NMDAR currents compared to seronegative controls [20]. Tajima et al. have reported recently that different expression-purified IgGs in mice immunised with intact NMDAR GluN1/GluN2A protein can produce differential effects on NMDA currents in oocytes, ranging from rapid block to increases or to no effect [18]. These data point to differential functional effects between heterogenous Aab components.

The lack of functional effects by anti-NMDAR Aabs here could be explained by a number of alternative factors. Anti-NMDAR Aabs may interact with signalling or scaffolding proteins, which are absent in the current oocyte system but present in native neurons. A candidate protein here is the ephrin-B2 receptor, which stabilises and clusters NMDARs at synaptic surfaces [25]. In cultured neurons, anti-NMDAR Aabs were shown to disrupt the GluN1 subunit interaction with ephrin-B2 receptors and prevented receptor internalisation [26]. Moreover, the co-administration of ephrin B2 together with CSF maintained NMDAR cell-surface levels and prevented pathogenic behavioural effects [27]. Therefore, oocytes might lack the mechanistic capabilities to reproduce the protein complexes required for such modulatory process [28].

Our data in oocytes show that anti-NMDAR Aabs lack any clear, direct effects on ionotropic NMDARs, such as channel block or negative allosteric modulation even after a 60 min incubation. Longer term incubations (>24 h) typically suppress excitatory postsynaptic currents (EPSCs) [7,29,30] and whole-cell NMDAR currents [31]. However, the acute application of different IgG species raised against NMDARs leads to differential effects, ranging from no effect to blockade or the potentiation of NMDAR currents. The acute application of patient CSF combined with an NMDAR agonist increased the mean open time of recombinant NMDARs in single channel recordings [24]. It was also reported that a 30 min application of patient CSF had no effect on NMDAR-mediated mEPSCs in hippocampal neurons; by contrast, receptor internalisation was seen after 12 h [15]. This study concluded that patient CSF effects occurred independently of NMDAR antagonisms and are due to longer term receptor internalisation. Related to this, it was reported that monoclonal antibodies from ANRE patients lacked effects on miniature spontaneous calcium transients in hippocampal neurons in response to a 10 min exposure [32]; by contrast, similar monoclonal antibodies could bind NMDARs in hippocampal neurons and be internalised over a 45 min timeframe [17]. Thus, a hypothesis arises whereby heterogenous anti-NMDAR Aabs may engage different modes of actions, namely, an ionotropic effect on NMDAR channels and/or receptor internalisation; moreover, such effects may be time-dependent for selected species.

### 3.3. Anti-NMDAR Aabs Suppress LTP 

Anti-NMDA Aabs produced by peptide purification had a significant inhibitory effect on HFS-induced potentiation in MEA-LTP experiments. Effects of anti-NMDAR Aabs were of similar magnitude to the NMDAR antagonist APV. Our data are in general agreement with previous studies, whereby exposure to patient CSF-containing anti-NMDAR Aabs for 1–8 days prior to testing caused a consistent, significant inhibition of LTP across several different pathways within the hippocampus [29,33,34]. Blome et al. (2018) further reported substantial variation among CSF samples [33]; these observations support the presence of different epitopes among patient-derived antibodies. Together, these studies are proposed to reflect a reduction in the synaptic density of NMDARs due to receptor internalisation. Studies using advanced single-particle tracking to investigate receptor internalisation demonstrated differential effects of patient CSF, whereby synaptic GluN2A was removed from the surface, but extrasynaptic GluN2B was mainly cross-linked by Aabs [26]. By contrast to the majority of studies above, an ‘acute’ 5 min pre-incubation was reported to cause a significant inhibition of LTP, suggesting a direct receptor block rather than an internalisation process [35]. Mikasova et al. (2012) investigated a time course of action, reporting that patient CSF caused a significant reduction in NMDAR cell surface localization after 2 h, which became greater after 20 h of incubation [26]. As discussed above, such studies are consistent with some species of anti-NMDAR Aabs being able to mediate an acute and/or longer-term action on NMDARs.

Neither of the two commercial antibodies or class-specific negative controls had effects on LTP here, the latter arguing against any non-NMDAR-specific IgG effects. Würdemann et al. (2016) reported that patient CSF-induced reductions in LTP were similarly shown by a commercial antibody raised against cytoplasmic C terminal amino acid (aa) 909-938 [29], also used by Hughes et al. (2010) [7]; however, it is unclear how this internal region can be accessed in these experiments. Würdemann et al. (2016) further reported that commercial GluN1 or GluN2 antibodies reduced LTP [29]; however, these were directed against different sequences to the ones targeted by the commercial antibodies used here. Overall, it was of interest that, despite the immunocytochemical labelling of NMDARs, neither of the commercial antibodies had functional effects in ex vivo slices, nor were any functional effects seen for mNMDARs in oocyte experiments. These commercial antibodies are directed against single target epitope sequences within the ATD of the GluN1 subunit (mNMDAR: aa660-811; rNMDAR: aa35-53); these sequences differ to those on which we based our five peptides used to generate anti-NMDAR Aabs. These data further suggest that, in mechanistic studies, it is important to target residues of functional importance. The data may also reflect that a strategy involving the use of multiple immunising peptides and/or cyclo-peptides to generate Aabs is advantageous to detect specific functional effects.

### 3.4. Clinical Relevance

It can be hypothesised that our anti-NMDAR Aabs caused NMDAR hypofunction, potentially via a mechanism involving receptor internalisation, rather than a direct block of NMDAR channel gating. The consensus view is that anti-NMDAR Aabs present in patient CSF is deleterious and pathogenic in different diseases including, but not limited to, ANRE and autoimmune epilepsies [36,37,38]. It is also broadly recognised that NMDAR hypofunction causes the seizures associated with such diseases, due to the disruption of neuronal circuitry and changes in excitatory/inhibitory balance [30,39]. The mainstay of treatment options for disorders such as ANRE are plasma exchange, intravenous immunoglobulins and steroids, with second-line agents including immunosuppressants such as rituximab [40,41]. There is also good pharmacological evidence that positive allosteric modulators may be useful to improve the receptor hypofunction caused by anti-NMDAR Aabs [31,42,43]. Recent advances in engineering immunological elements may also bear fruit in new therapies. Thus, anti-NMDAR Aab-mediated NMDAR internalisation can be prevented by a fusion construct comprising the IgG Fc region and GluN1 and GluN2 subunit ATDs [44]. It has also been reported that the neonatal Fc receptor can prevent the patient sera-induced inhibition of LTP [45]. As discussed above, patient CSF and/or sera may contain many different component Aabs to different receptors and NMDAR Aabs may exhibit heterogenous effects, some of which might appear contradictory to those of NMDAR antibodies designed as therapeutic agents [46]. Therefore, it is important to develop appropriate tools, such as the peptide-purified anti-NMDAR Aabs investigated here, with which to investigate NMDAR physiology and potential pathophysiology, in order to best develop new therapies strategies to combat devastating NMDAR-associated diseases. 

## 4. Materials and Methods

### 4.1. Antibody Design, Production and Purification

Five peptides were designed by UCB and Peptide Synthetics, UK to target amino acid (aa) sequences in the extracellular loops of the human GluN1 (NR1) subunit (GenBank accession Q05586): (1) aa125-135, MSIYSDKSIHL; (2) aa249-266, cyclo-VGEREISGNALRYAPDGI; (3) aa272-281, INGKNESAHI; (4) aa353-363, cyclo-IMNLQNRKLVQ; and (5) aa365-375, cyclo-GIYNGTHVIPN (see Figure 1A). All peptides had N-terminal acetylation and C-terminal amidation. Peptides 2, 4 and 5 were cyclised via a thioester to aid in mimicking the natural 3D structure of their respective epitopes [47], thereby increasing the chances of generating Aabs that bind to the GluN1 subunit in its natural conformation.

Antibodies were raised using the 5 peptides (500 µg of peptide per immunisation) with booster injections at 14-day intervals in one female New Zealand White rabbit (>2 kg), as described previously [48]. The rabbit was sacrificed 14 days after the final immunisation by Schedule 1 methods in accordance with the UK Animals (Scientific Procedures) Act 1986 and the terminal serum collected. 

Protein A-purified Aabs (12 mg/mL) were prepared as described previously [48] and further purified through a high-capacity streptavidin agarose resin (Pierce; Thermo Fisher Scientific, Loughborough, UK). Biotin-bound peptides (peptides 1–5) used for immunisation (133 μM each) were first mixed with a 5 mL streptavidin agarose resin. This was then combined with Protein A-purified Aabs (overnight at 4 °C), applied to columns, washed with PBS and a peptide-specific antibody eluted with a concentration of 0.1 M sodium citrate (pH 3.2). Pooled fractions were pH-neutralised with Tris-HCl (pH 8.5). Fractions were concentrated through a buffer exchange with PBS using 10 kDa of MWCO. The total peptide-specific IgG concentration (1.3 mg/mL) was determined using absorbance at 280 nm, SDS-PAGE and ELISA, as described previously [48].

### 4.2. Expression of NMDARs in HEK Cells 

HEK cells were grown in DMEM + 10% FBS media. Cells were transfected with plasmids encoding the GluN1 (NR1) subunit (pcDNA3.1(+) NR1-4a_HS) and GluN2b (NR2B, pcDNA3.1(−) NR2B_HS), respectively; control cells were transfected with an empty plasmid vector; pcDNA3.1(+). Transfections used a 2:1 ratio polyethylenimine (PEI):DNA inOptiMem (Thermo Fisher Scientific) for 6 h before sub-culture onto poly-D-lysine-coated coverslips (PDL; 20 μg/mL; Sigma-Aldrich (Merck), Gillingham, UK) at 2.5 × 10^5^ cells/well for 24 h. Cells were then fixed with 4% PFA for 10 min (Sigma-Aldrich) before processing for immunocytochemistry. 

### 4.3. Animals

The housing and use of animals in all experiments were carried out in accordance with UK Home Office regulations under the Animals (Scientific Procedures) Act, 1986. Mice were housed at 21 °C in a 12 h light/dark cycle with food and water available ad libitum according to ARRIVE guidelines [49].

#### 4.3.1. Preparation of Acute Mouse Hippocampal Slices 

Transverse hippocampal slices of 400 μm thickness were prepared from male 4–6-week-old C57BL6/J mice. Mice were terminally anaesthetised with 4% isoflurane and immediately underwent cervical dislocation and decapitation. Slices were produced using a high-sucrose artificial cerebrospinal fluid (aCSF) cutting solution, comprising (in mM) the following: sucrose (75), NaCl (87), NaHCO_3_ (25), KCl (2.5), NaH_2_PO_4_ (1.25), CaCl_2_ (0.5), MgCl_2_ (7) and glucose (25), pH 7.4, (all Thermo Fisher Scientific) on a VT1200S vibrotome (Leica) and transferred to carboxygenated aCSF, comprising (in mM) the following: NaCl (126), glucose (10), MgCl_2_ (2), KCl (2.5), NaH_2_PO_4_ (1.25), NaHCO_3_ (26) and CaCl_2_ (0.5), pH 7.4, at 37 °C for 30 min. Slices were equilibrated at room temperature for at least 1 h before use.

#### 4.3.2. Embryonic Day18 (E18) Mouse Primary Neuronal Cell Culture

Cortical cultures were prepared as described previously [48]. Briefly, primary neuronal cultures were prepared from male and female E18 C57BL/6 mice as a model of NMDAR expression, although we appreciate that sex is an important biological variable [50] and that using mixed sex cultures might represent a limitation here. After the removal of meninges, dissected cortices were chemically dissociated using papain and DNase (Sigma-Aldrich) and diluted in a culture medium (Neurobasal medium with 1% B27, 2 mM of GlutaMax, 2.5% FBS and 100 U/mL/100 µg/mL of penicillin/streptomycin; all Life Technologies, Thermo Fisher Scientific). Cells were pelleted and resuspended in a culture medium before plating at 2 × 10^5^ cells/well on laminin-coated coverslips (Sigma-Aldrich) in 24-well plates. A 50% culture medium change was conducted every 2–3 days. Cells were washed with PBS and fixed with 4% PFA for 10 min (Sigma-Aldrich) before processing for immunocytochemistry. 

### 4.4. Immunocytochemistry and Immunoblotting

Primary and secondary antibodies used were as follows: rabbit anti-NMDAR (rNMDAR: 1:100, polyclonal raised against residues 35–53 of human GluN1, Synaptic Systems, Cat No. 114 103,); mouse anti-NMDAR (mNMDAR: 1:100, monoclonal raised against residues 660-811 of human GluN1, Synaptic Systems, Cat No. 114 011); rabbit anti-IgG (rIgG, 1:100, 011-000-003, Jackson ImmunoResearch, Ely, UK), 11.1 mg/mL); mouse anti-IgG2 (mIgG2b, 1:100, 70-4732, BioLegend, London, UK); mouse anti-βIII-tubulin (1:500, 801201, BioLegend); mouse anti-glial fibrillary acidic protein (GFAP, 1:400, MAB3402, Millipore, Merck, Gillingham, UK); and goat anti-rabbit or anti-mouse Alexa Fluor 488/594/647 (all at 1:1000, Life Technologies, Thermo Fisher Scientific). Transfected HEK cells were processed as described previously [48].

#### 4.4.1. Immunocytochemistry on Primary Neurons 

Cortical neurons were used as a GluN1-expressing cell model. Immunocytochemistry was performed essentially as described in [47]. Briefly, following fixation, cells were incubated for 2 h at room temperature with primary antibodies in blocking buffer (10% normal goat serum in PBS) either before permeabilisation (anti-NMDAR Aabs, rIgG, or mIgG2b) or after permeabilisation (anti-βIII tubulin or anti-GFAP) with 0.1% Triton-X in PBS, followed by a 30 min incubation with Alexa Fluor-conjugated secondary antibodies. Cells were mounted and imaged as described previously [48].

#### 4.4.2. Immunoblot of Brain Lysates

SDS-PAGE and Western blotting were performed as described previously [48]. Briefly, protein lysates were prepared from mouse whole brain tissue (C57BL6/J, male 4–6 weeks) using the lysis buffer of NaCl (150 mM), Triton-X-100 1% (*v*/*v*) glycerol 10% (*v*/*v*), HEPES (30 mM), and SigmaFAST protease inhibitors (1 tablet/50 mL). Protein concentration was determined using a bicinchoninic acid protein assay kit (Thermo Fisher Scientific). SDS-PAGE gels were prepared with a 10% separating gel and 3% stacking gels. Western blotting was used to assess the specificity of NMDAR Aabs against protein lysate.

### 4.5. Xenopus Oocyte Expression System

GRIN1/GRIN2A constructs encoding GluN1 and GluN2A were generated, prepared and expressed in *Xenopus* oocytes (stage V–VI) as described previously [21].

#### 4.5.1. Two Electrode Voltage Clamp (TEVC) Electrophysiology in *Xenopus* Oocytes 

TEVC recordings were performed using an automated platform (HiClamp^®^, MCS, Dinslaken, Germany). Electrodes (0.1–1 MΩ resistance) were filled with potassium chloride (KCl, 1.5 M) and potassium acetate (Kac, 1.5 M). Oocytes were impaled and voltage-clamped at a holding potential of −80 mV. Oocytes were then rinsed, and recordings for the glutamate response were performed in modified frog buffer, comprising (in mM) the following: NaCl (88), KCl (2.5), CaCl_2_ (1.8) and HEPES (5), pH 7.85, at 19 °C as described previously [21]. Oocytes were pre-incubated for 3 min in the control buffer, and currents were induced by 10 s applications of 1 or 10 μM of glutamate/10 μM of glycine (glu/gly) every 3 min for 12 min to generate stable NMDAR-mediated currents, then incubated in the test substance and challenged by 10 s applications of glu/gly every 15 min (total exposure to antibodies = 60 min). In different oocytes, the NMDAR negative allosteric modulator TCN-201 (Sigma-Aldrich, 0.01–3 µM) was used as a positive control to show the inhibition of currents. Current responses were measured by the AUC and normalised to the AUC from control responses.

#### 4.5.2. Data Analysis Oocytes

Currents were analysed off-line using the HiClamp Software V01-7.6 (DataMining and DataMerger) as described previously [21]. 

### 4.6. MEA Recordings

Evoked electrical activity was recorded in ex vivo hippocampal brain slices using titanium nitrate MEAs (MCS) via head stage (MEA1060-Inv-BC, MCS), whereby biphasic current pulses applying the negative phase first were applied to one electrode (STG2008 stimulator, MCS; 100 μs biphasic pulses, ±0.5–2.0 V, every 30 s) to evoke fEPSPs [51]. Signals were amplified by a 60-channel head-stage amplifier (MEA60 System, MCS) and simultaneously sampled at 10 kHz per channel and amplified at 1200× gain. Data acquisition to a computer was carried out using the software MC_Rack V4.6.2, which monitored and recorded data for later offline analysis. Slices were maintained at 32 °C and perfused with carboxygenated aCSF (~3 mL/min).

Schaffer collateral-CA1 LTP was induced using HFS: protocol was 100 Hz to ±1000 mV for 1s, repeated 4× total (every 20 s). Paired pulse fEPSPs were evoked every 30 s and recorded for 30 min pre-LTP induction to establish a steady baseline and for 60 min post-LTP induction. HFS potentiation was measured as changes to the fEPSP slope as a measure of excitatory drive. The fEPSPs could be isolated into separate glutamatergic components: AMPAR/kainate receptors or NMDARs were blocked by the addition of 6-cyano-7-nitroquinoxaline-2,3-dione (CNQX 5 μM; Abcam, Cambridge, UK) or DL-2-amino-5- phosphonopentanoic acid (APV 50 μM; Abcam), respectively, to the perfusing aCSF. Slices were pre-incubated for 1 h in aCSF containing anti-NMDAR Aabs or IgG controls (both 1:1000 dilution) with continuous carboxygenation. Slices were also incubated with either mNMDAR, rNMDAR or mIgG2b/rIgG antibodies as positive and negative controls, respectively, (1:1000 dilution). One slice per animal was used per condition.

### 4.7. Statistical Analysis 

All data are presented as mean ± standard deviation with the number of independent experiments (n) detailed and analysed using GraphPad Prism 7.00. All data sets were tested for normality using D’Agostino Pearson tests; on this basis, parametric one- or two-way ANOVAs with Dunnett’s multiple comparison post hoc tests were performed. Throughout, data were considered significant at *p* < 0.05. 

## Data Availability

Data are contained within this article, and raw data presented in this study are available upon request.

## Supplementary Materials

The following supporting information can be downloaded at: https://www.mdpi.com/article/10.3390/ph17121643/s1, Figure S1: Immunocytochemistry staining controls. Figure S2. Protein A-purified anti-NMDAR Aabs have no effect on NMDA current in *Xenopus* oocytes.

## Abbreviations

Autoantibodies (Aabs), artificial cerebrospinal fluid (aCSF), α-amino-3-hydroxy-5-methyl-4-isoxazolepropionic acid (AMPA), amino terminal domain (ATD), anti-NMDAR encephalitis (ANRE), DL-2-amino-5-phosphonopentanoic acid (APV), area under the curve (AUC), 6-cyano-7-nitroquinoxaline-2,3-dione (CNQX), central nervous system (CNS), cerebrospinal fluid (CSF), embryonic day18 (E18), equilibrative nucleoside transporter 1 (ENT1), excitatory postsynaptic currents (EPSCs), field excitatory postsynaptic potential (fEPSP), glutamate/glycine (glu/gly), human embryonic kidney 293 (HEK), high-frequency stimulation (HFS), long-term potentiation (LTP), mouse monoclonal anti-GluN1 antibody (mNMDAR), multi-electrode array (MEA), N-methyl-D-aspartate receptor (NMDAR), rabbit polyclonal anti-GluN1 antibody (rNMDAR).
