# Supplementary material for: Peptide-Purified Anti-N-methyl-D-aspartate Receptor (NMDAR) Autoantibodies Have Inhibitory Effect on Long-Term Synaptic Plasticity
Source: Pharmaceuticals (Basel). 2024 Dec 6;17(12):1643. doi: 10.3390/ph17121643 (PMC11677035; doi:10.3390/ph17121643)
Supplement: Supplementary file 1 [file pharmaceuticals-17-01643-s001.zip › pharmaceuticals-3219691-supplementary.pdf]

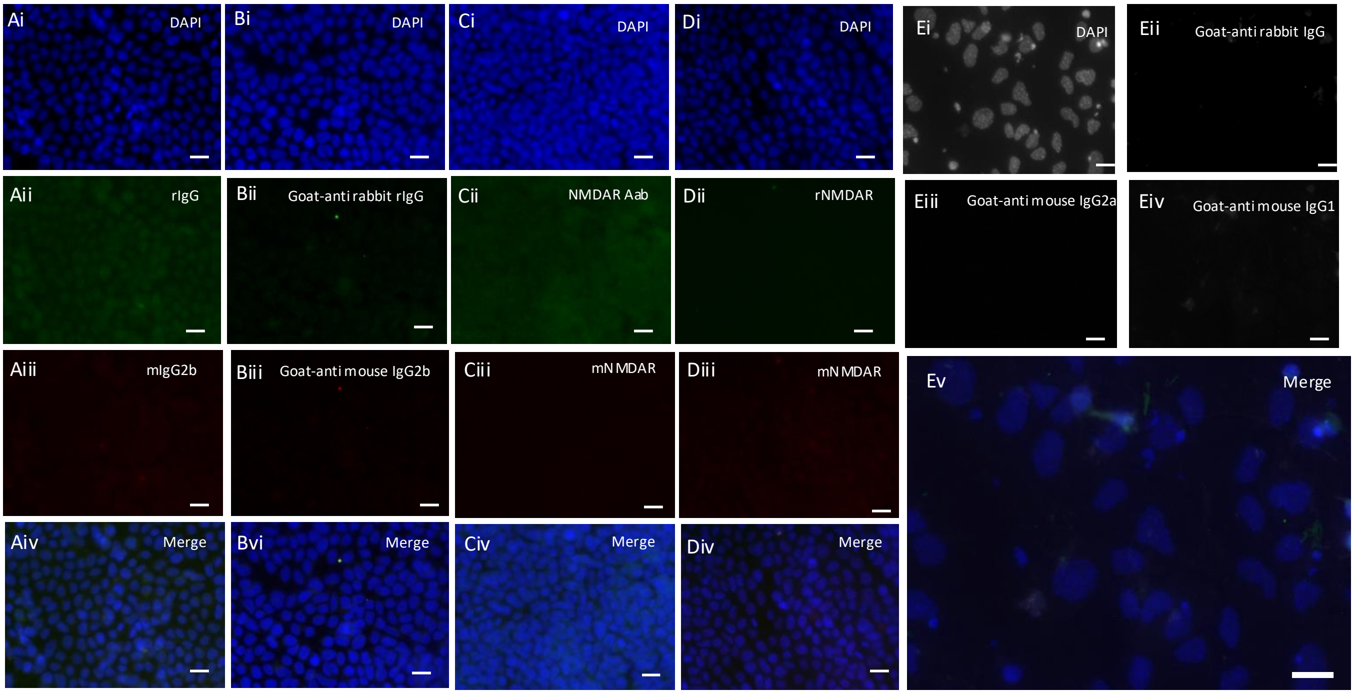

Supplemental Figure 1: Immunocytochemistry staining controls.
GluN1-transfected HEK cells incubated with **(Ai-Aiv)** class-specific negative controls rIgG and mIgG2b or **(Bi-Biv)** secondary-only antibody controls showed no positive staining. **(Ci-Civ, Di-Div)** Empty vector-transfected HEK cells showed no clear staining when incubated with anti-NMDAR1 Aabs (1:100) or with each one of two commercial anti-NR1 antibodies (1:100). Representative image selected from n=3 biological replicates. **(Ei-Ev)** Primary cortical neuronal cells (DIV14) incubated with the secondary-only antibody control showed no staining in any channel. Representative image selected from n=3 biological replicates.
Scale bar = 20 µm throughout.

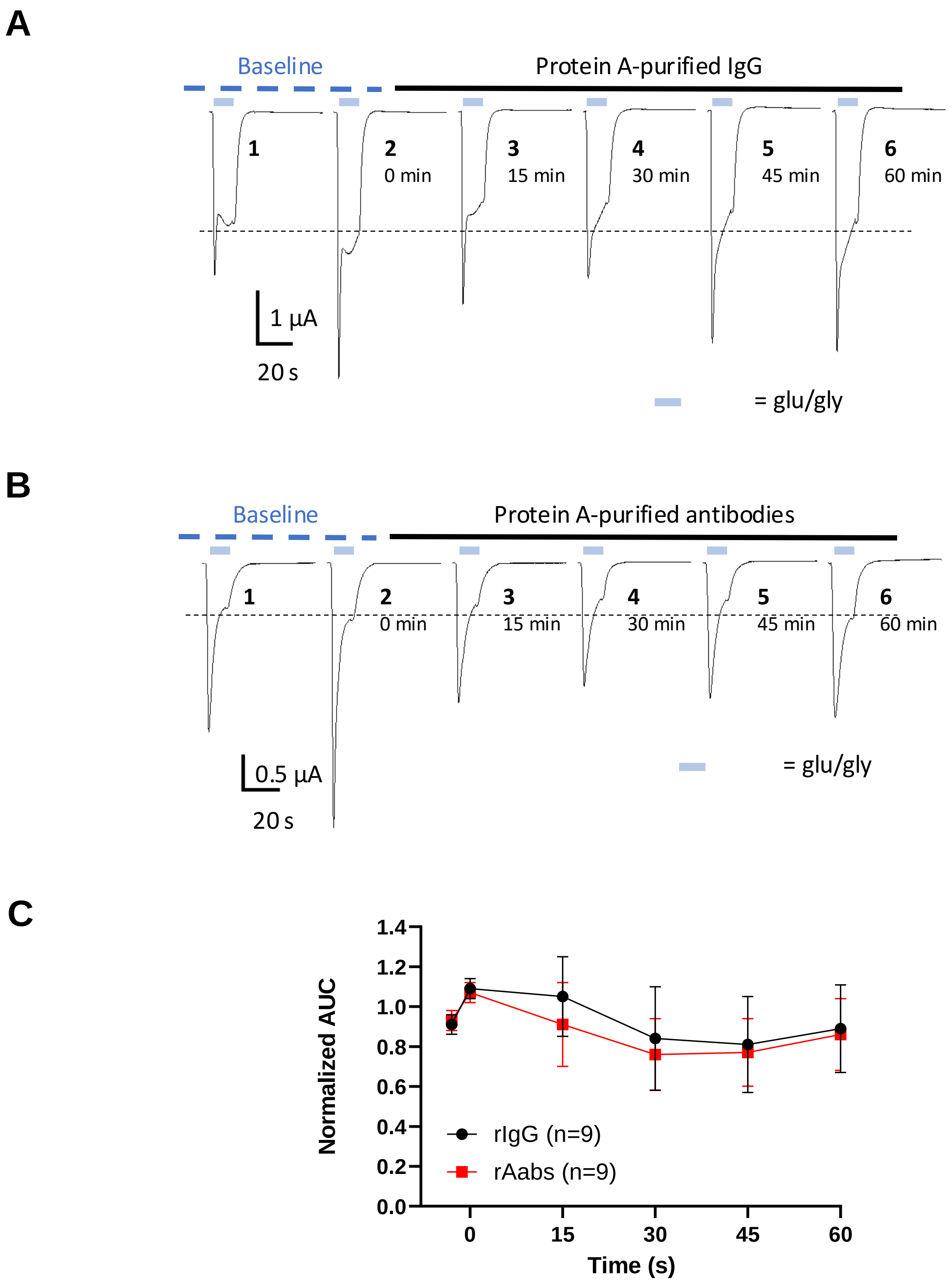

Supplemental Figure 2. Protein A-purified anti-NMDAR Aabs have no effect on NMDA current in Xenopus oocytes.
Traces of NMDAR-evoked responses over time before and after incubation of Protein A-purified antibodies: 1 µM glutamate/10 µM glycine-induced NMDA currents were elicited every 15 min in the presence of **(A)** Protein A-purified IgG or **(B)** Protein A-purified anti-NMDAR Aabs (both 1:300 dilution) applied for up to 60 min. **(C)** Graph shows effect of Protein A-purified anti-NMDAR Aab and rIgG glutamate-evoked (AUC) responses normalised to baseline (mean of applications 1 and 2 shown above). There was no significant change in current AUC when compared to baseline in anti-NMDAR Aabs or rIgG incubated oocytes. Each oocyte was used for baseline and treatment throughout the 60 min experiment.
